# Use of live yeast and mannan-oligosaccharides in grain-based diets for cattle: Ruminal parameters, nutrient digestibility, and inflammatory response

**DOI:** 10.1371/journal.pone.0207127

**Published:** 2018-11-14

**Authors:** Tatiana Garcia Diaz, Antonio Ferriani Branco, Fernando Alberto Jacovaci, Clóves Cabreira Jobim, João Luiz Pratti Daniel, Antonio Vinicius Iank Bueno, Matheus Gonçalves Ribeiro

**Affiliations:** Department of Animal Science, State University of Maringá; Bloco J45, Maringá, PR, Brazil; University of Illinois, UNITED STATES

## Abstract

The objective of this study was to evaluate the effects of diet supplementation with live yeast (*Saccharomyces cerevisiae*) and mannan-oligosaccharides (MOS) on ruminal parameters, nutrient digestibility, and the inflammatory response in cattle fed grain-based diets. Three Holstein steers (body weight of 497±3 kg) with ruminal and duodenal cannulas were assigned to a 3 × 3 Latin square design. The animals were kept in individual pens and fed a diet containing 5% sugarcane bagasse and 95% concentrate (906.5 g/kg ground corn). Diet treatments were Control (without additive), Yeast (1.5 g/kg DM live yeast *Saccharomyces cerevisiae*, NCYC 996) and MOS (1.5 g/kg DM MOS, β-glucans and mannan). Dry matter intake, ruminal, intestinal, and total digestibility of nutrients were not affected by the treatments. The ruminal concentration of isobutyric acid increased in animals fed on diets supplemented with Yeast and MOS, whereas isovaleric acid increased with Yeast and decreased with MOS supplementation. Dietary supplementation with Yeast and MOS increased pH and decreased ammonia concentration in the rumen. Lipopolysaccharide (LPS) concentrations in the rumen and duodenal fluid were not influenced by the additives. However, both Yeast and MOS decreased the plasma levels of LPS and serum amyloid A (SAA). In conclusion, when high-concentrate diets fed to beef cattle are supplemented with either Yeast or MOS, ruminal pH is increased, LPS translocation into the blood stream is decreased, and blood SAA concentration is decreased. These factors reduce the inflammation caused by consumption of grain-based diets, and either supplement could be used to improve the rumen environment in beef cattle susceptible to ruminal subacute acidosis.

## Introduction

Direct-fed microbials (DFMs) are defined as naturally occurring live microorganisms that are used to improve the digestive function of animals [1, 2, 3, 4]. Microorganisms used as DFMs for ruminants include live yeast (*Saccharomyces cerevisiae)* known for their health and animal performance benefits [2, 3, 5]. Mannan-oligosaccharides (MOS) are prebiotics composed of complex carbohydrate molecules, derived from the outer cell wall of *S*. *cerevisiae*, whose main components, β-glucans (mannoproteins), are known as elements capable of activating the immune system of animals [3, 5].

Some of the main benefits of yeast and MOS mentioned in the literature are related to the potential to improve animal performance [6], prevent lactate accumulation in the rumen [7], stabilize rumen pH [8, 9], and decrease the production and absorption of toxic molecules [8, 10]. Thus, these additives may help to improve not only the performance of cattle, but also nutrient digestibility [2, 11, 12]. For these reasons, both probiotics and prebiotics are added to ruminant feed in order to prevent digestive disorders, especially those related to a high-energy intake in finishing beef cattle, such as subacute ruminal acidosis [9, 10, 13].

However, as observed in many studies so far, the effectiveness of dietary yeast products is variable. Therefore, further investigation is needed to evaluate the potential of these products as additives for finishing beef cattle, aiming to attain healthier and more productive animals without compromising efficiency and costs. The objective of this study was to evaluate the effects of supplementing live yeast (*S*. *cerevisiae*) and MOS on ruminal parameters, nutrient digestibility, and the inflammatory response in cattle fed a high-grain diet.

## Material and methods

The experiment was conducted at the Iguatemi Experimental Research Farm, State University of Maringá (UEM), Maringá, PR, Brazil. All procedures were performed in accordance with Brazil’s National Council for the Control of Animal Experimentation (CONCEA) guidelines and were authorized by the Ethics Committee for Animal Use of the State University of Maringá, PR, Brazil (Protocol number: 6479060416).

### Animals, experimental design and treatments

Three Holstein steers (body weight of 497±3 kg) with ruminal and duodenal cannulas were assigned to a 3 × 3 Latin square design, with a duration of 21 days in each period, consisting of 14 days for adaptation and 7days for sampling. The animals were housed at the Sector Ruminants Nutrition of the Iguatemi Experimental Research Farm in covered stalls with concrete floors (2.5 x 3.5 m), individual feed bunkers and automatic waterer.

Diets contained 5% sugarcane bagasse and 95% concentrate (ground corn, urea, and mineral mixture). Diet treatments were Yeast (1.5 g/kg DM of live yeast *Saccharomyces cerevisiae* NCYC 996, Procreatin-7, Phileo LeSaffre Animal Care, Campinas, Brazil) MOS (1.5 g/kg DM of MOS, β-glucans and mannan, Safmannan, Phileo LeSaffre Animal Care, Campinas, Brazil) and Control (without additive), this treatment received 1.5 g/kg of calcium carbonate to ensure that the balance of other ingredients in the diet was similar. The viability of live yeast was checked prior to starting the experiment using Yeast Extract-Peptone-Dextrose (YPD) Agar and incubated at 30°C for 3 days. The mean number of Yeast colonies detected on agar were 2.74 ± 0.83 × 10^10^colony forming units (CFU)/g for live yeast.

### Experimental procedures

Before the beginning of the experiment, the animals were adapted to the Control diet in a step-up system for 15 days. During the adaptation process, the diets were consumed ad libitum and had levels of concentrate (55; 65 and 75%) increased every 5 days up to the defined percentage of 95%. The experiment was started after 15 days of adaptation.

The total mixed rations were offered *ad libitum* twice daily at 08h00min and 16h00min to allow 10% of orts. Samples of orts were collected in plastic bags across 7 days, from the 15th day of each experimental period. Samples of feeds (corn and sugarcane bagasse) were collected three times weekly and composited. Dry matter (DM) was determined on a portion of each weekly composite feed sample, and the DM contents were used to adjust the and sugarcane bagasse to concentrate ratio of the diet when necessary. Samples were dried, and DM intake (DMI) for each steer was calculated based on the feed DM offered and orts DM refused.

From the 7th day of each experimental period, 10 g of titanium dioxide (TiO_2_) was administered directly into the rumen in the morning as the external marker for the evaluation of duodenal flow and fecal excretion. The ruminal and total-tract digestibility of DM, organic matter (OM), crude protein (CP), neutral detergent fiber corrected for ash and protein (NDFap), acid detergent fiber (ADF), and non-fiber carbohydrates (NFC) were determined by sampling duodenal digesta (500 mL) and fecal samples (50 g). These samples were collected across 3 days (on the 15th through the 17th day of each experimental period) at different times (08:00, 14:00, 20:00, 02:00, 10:00, 16:00, 22:00, 4:00, 12:00, 18:00 and 24:00 and 06:00 h), totaling 12 samples of duodenal digesta and 12 fecal samples per animal/period.

Samples of feeds (corn and sugarcane bagasse), orts, feces, and duodenal digesta were frozen at -20° C. At the end of each collection period, the samples were thawed, homogenized, and dried in a forced-air oven at 55°C for 72 h. Subsequently, the samples were ground individually in a Wiley mill equipped with a 1 mm screen. Then, the samples were mixed in the same ratio (10% of the sample based on the dry weight) to form samples composed of orts, feces, and duodenal per animal/period/treatment. The results of each nutrient of the feeds that compose the diet were multiplied by the percentage of inclusion of each food in the diet and described in Table 1.

**Table 1:** The composition of experimental diets (g/kg dry matter).

Item	Diet
Ingredient	
Sugarcane bagasse	50.0
Ground corn	906.5
Mineral mix^1^	9.0
Urea	18.0
Salt	2.0
Limestone	13.0
Treatments ^2^^,^ ^3^^,^ ^4^	1.5
Chemical composition [g/kg as fed]	
DM^5^	819
CP^6^	133
NDF_ap_^7^	149
ADF^8^	66
NFC^9^	697
NEm^10^ (Mcal/kg DM)	1.94
NEg^10^ (Mcal/kg DM)	1.37

^1^Mineral mix: Elemental sulfur 70S: 141.2 mg/kg; Calcium iodate: 0.484 mg/kg; Magnesium oxide: 320.2 mg/kg; Sodium selenite: 0.105 mg/kg; Cobalt sulfate: 0.400 mg/kg; Copper sulfate: 19.64 mg/kg; Manganese sulfate 10.76 mg/kg; Zinc sulfate: 13.75 mg/kg.

^2^Control (without additive, 1.5 g/kg DM of calcium carbonate).

^3^Yeast (1.5 g/kg DM of live yeast *S*. *cerevisiae*)

^4^MOS (1.5 g/kg DM of mannan-oligosaccharides).

^5^Dry matter (DM).

^6^Crude protein (CP).

^7^Neutral detergent fiber corrected for ash and protein (NDF_ap_).

^8^Acid detergent fiber (ADF).

^9^Non-fibrous carbohydrate (NFC).

^10^Net energy for maintenance (NEm) and for gain (NEg).

On the 15th day, samples of ruminal fluid (150 mL) were collected at 0, 3, 6, 9, and 12 h after the morning feeding for analysis of short-chain fatty acids (SCFA) and at 0, 3, 6, 9, 12, 15, 18, and 21 h after the morning feeding for analysis of ammonia (NH_3_). Ruminal fluid samples obtained from different sites within the rumen (dorsal and ventral sacs) using a suction-strainer device, compound of a PVC tube (48 cm long × 1.5 cm o.d. × 1.2 cm i.d.) with holes in it, a flask, and a manual suction pump. For the determination of NH_3_, a 50 mL sample of ruminal liquid was acidified with 1 mL of 50% (vol/vol) sulfuric acid (H_2_SO_4_) and for the determination of SCFA, a 10 mL were acidified samples with 2 mL of 25% (weight/volume) metaphosphoric acid and stored at −20°C until later analyses.

On the 15th and 17th day of each experimental period, samples of 50 mL of ruminal fluid were collected every hour for measuring the pH over 24 h. Ruminal fluid samples were obtained from different sites within the rumen (dorsal and ventral sacs) using a suction-strainer device. The pH was measured immediately after each sampling using a digital pHmeter calibrated with pH 4.0 and pH 7.0 standards.

On the 18th and 20th day of each experimental period, blood samples were drawn by jugular puncture using 10 mL vacuum tubes containing heparin, 15 minutes before the morning feeding (07:45 h) and then at 6 and 12 h after the first feeding. The blood was centrifuged at 3000 x *g* for 10 min to separate the plasma, which was stored in Eppendorf tubes at -20°C for further analysis of LPS, SAA, and haptoglobin (Hp). On the same days and times, ruminal fluid samples (15 mL) and intestinal digesta (15 mL) were collected for LPS analysis. They were transferred into sterile tubes and centrifuged at 6000 x *g* for 15 min at 4°C, as described by Khafipour et al. [14] and Emmanuel et al. [15]. The supernatant was stored at -20°C for analysis of LPS concentration.

On the 19th and 21st day of each experimental period, blood samples were collected by jugular puncture using vacutainer tubes containing EDTA (Ethylenediamine tetra acetic acid), before the first morning feeding. The blood samples collected were stored in Styrofoam boxes with dry ice and sent immediately for analysis sent to São Camilo Group—Veterinary Division, Maringá, PR, Brazil for measuring erythrogram and leukogram patterns.

### Chemical analysis

Samples from the experimental diets, duodenal digesta, feces and orts were analyzed for DM (method 934.01), ash (method 942.05), CP (method 984.13), and EE (945.16, Soxhlet extraction with petroleum ether) according to the methodologies proposed by AOAC [16]; while ADF and NDF, corrected for ash and protein, were analyzed by using neutral detergent insoluble nitrogen (NDIN, without sodium sulphite) following the method of Van Soest et al. [17] with addition of heat stable α-amylase. Duodenal digesta and feces were analyzed for titanium according to Myers et al. [18]. Non-fibrous carbohydrate was calculated according to the NRC [19]. Net energy for maintenance (NEm) and for gain (NEg) were estimated according to the NRC [20].

Rumen fluid samples preserved with sulfuric acid were thawed and centrifuged at 3,000 × g for 15 min at 4°C and analyzed for NH_3_ using a technical colorimetric method of Chaney and Marbach [21]. Rumen fluid samples that were metaphosphoric acid were thawed and centrifuged at 30,000 × *g* for 20 min at 4°C and analyzed while the concentration of SCFA was determined by using a gas Chromatograph (SHIMADZU, model GC-2014) equipped with an automatic injector (model AOC-20). The injector temperature was 200°C, and the column temperature was 80°C/3 min till 240°C. The column used was HP INNOwax - 19091N (30 m long, 0.32 mm ID, 0.50 μm film) and the detector was flame ionization.

The SAA and Hp were determined with commercial kits, Tridelta SAA Multispecies Assay (Induslab, Londrina, PR, Brazil) and Tridelta PHASE Haptoglobin Multispecies Assay (Induslab, Londrina, PR, Brazil), respectively. The analytical sensitivities of these tests in serum have been determined as 1.5 μg/mL for SAA and 0.05 μg/mL for Hp by the manufacturer. The LPS in plasma, ruminal fluid, and duodenal digesta were determined by the chromogenic end-point assay using with the commercial kit, ToxinSensor chromogenic LAL Endotoxin Assay, GenScript (FastBio, Riberão Preto, SP, Brazil). The minimum detection level of LPS in the plasma was 0.01 EU/mL. The procedures followed the manufacturer's instructions.

### Statistical analysis

Data were analyzed using the MIXED procedure of the SAS statistical package [22]. Treatment (Control, Yeast and MOS) and period were considered fixed effects. Animal was considered a random effect. Day was considered a repeated effect for the variables. The models used for SCFA, LPS, SAA, and Hp also included the fixed effects of hours and the interactions of hours with the fixed factors.

Data for ruminal pH were summarized by day and then analyzed using the same mixed model procedure (using of SAS) with day included as a repeated measure and using compound symmetry. Ruminal pH data and NH_3_ mean, minimum, and maximum values of pH were calculated using the mixed model. The duration of pH below 5.8 and the area under the pH curve (AUC) were calculated as the sum of the absolute values of pH deviations under the curve, considering a limit of pH 5.8 expressed as pH × h d^-1^ as described by Vyas et al. [9]. Ruminal pH of 5.8 was chosen as an indicator of subacute ruminal acidosis since cellulolytic bacteria growth decreases below this pH [23].

The restricted maximum likelihood method was used for estimating the variance components. The better time-series covariance structures were selected based on the lowest Akaike and Bayesian information criteria. Time-series covariance structures were modeled using the options of ante-dependence (SCFA and LPS), variance components (SAA, SCFA, NH_3_), and unstructured order one (Hp and pH). The PDIFF option adjusted by the Tukey method was included in the LSMEANS statement to account for multiple comparisons. Effects were considered significant at α = 0.05. Trends were discussed at α = 0.15.

## Results and discussion

### Feed intake and digestibility

Daily intake of DM, CP, NDFap, and EE were not affected (P>0.05) by the addition of Yeast or MOS (Table 2). In the same way, the additives did not change (P>0.05) the ruminal, intestinal and total tract apparent digestibility of DM, CP, NDFap, EE and TDN (Table 2).

**Table 2:** Intake, duodenal and fecal flows, ruminal (RAD), intestinal (IAD) and total-tract apparent digestibility (TTAD) of DM, CP, NDFap, and EE of fed grain-based diet for cattle supplemented with Yeast (live yeast *S*. *cerevisiae*) and MOS (mannan-oligosaccharides).

Item	Treatments			SEM^1^	P- value
	Control	Yeast	MOS		
Intake [g/d]					
DM	9,648	11,039	10,580	1,180	0.56
CP	1,280	1,463	1,418	157.1	0.57
NDF_ap_	1,424	1,585	1,532	14.5	0.76
EE	384	439	422	36.3	0.43
Duodenal flow [g/d]					
DM	2,383	2,231	2,614	65.6	0.18
CP	311	358	391	50.3	0.21
NDF_ap_	644	658	651	28.6	0.36
EE	101	105	125	5.7	0.19
Fecal Matter [g/kg]					
DM	1,707	1,683	1,616	103,8	0.70
CP	236	282	238	21.7	0.25
NDF_ap_	522	540	531	36.4	0.93
EE	33.9	49.3	42.0	4.7	0.20
RAD [g/kg]					
DM	750	796	752	27.6	0.33
CP	752	769	726	56.8	0.77
NDF_ap_	547	575	567	27.4	0.83
EE	740	762	699	29.7	0.46
IAD [g/kg]					
DM	283	251	385	37.8	0.23
CP	231	266	326	55.2	0.44
NDF_ap_	186	184	185	36.2	0.99
EE	635	537	636	67.9	0.41
TTAD [g/kg]					
DM	822	848	848	10.3	0.32
CP	814	804	833	22.9	0.66
NDF_ap_	633	654	646	32.8	0.89
EE	910	890	901	5.0	0.20

^1^SEM = standard error of the mean; Tukey test (α = 0.05).

The effects of Yeast and MOS on feed intake and digestibility are not reported to be inconsistent in the literature. Vyas et al. [9] reported no effect on DM intake and digestibility containing the addition of 4 g/d of active dried yeast (*S*. *cerevisiae*, 10^10^ CFU/g) or 4 g/d of killed dried yeast on heifers fed high grain diets (33.8% starch). On the other hand, Magrin et al. [24] observed an increase in DM intake by finishing Charolais bulls fed diets containing 70.3% of concentrate with 5 g/bull of *S*. *cerevisiae* CNCM I-1077 (1×10^10^ CFU/bull/d). Discrepancies among experiments depend on different interacting factors, such as the type of diet, animal species and physiological state [24, 25, 26]. In addition, the strain and dose of Yeast and MOS may also influence the response of animals supplemented with these additives on intake, feed efficiency, digestibility and performance [2, 25, 26, 27].

### Ruminal parameters

Supplementation with Yeast or MOS tended to increase (P<0.14) the total concentration of SCFAs but did not influence (P>0.05) the molar proportions of acetate, propionate and butyrate (Table 3). Andrighetto et al. [28] and later Křižova et al. [29] obtained a significant increase in the total concentration of SCFAs in the rumen of cows fed grain-based diets and supplemented with a Yeast culture. In the same way, Mutsvangwa et al. [30] indicate that Yeast supplementation may produce an increase in the ruminal fermentation rate and bacterial population when yeast cultures are added to the bull’s diet, but not a change in the ruminal fermentation pattern. The mechanisms of action of the Yeast or MOS to account for a higher SCFA concentration in yeast-fed animals are not fully understood but appear to be associated with an increase in activity of the anaerobic microflora [31].

**Table 3:** Ruminal concentration of short-chain fatty acids (SCFA) in cattle fed grain-based diets supplemented with Yeast (live yeast *S*. *cerevisiae*) and MOS (mannan-oligosaccharides).

Item	Treatments			SEM^1^	P- value		
	Control	Yeast	MOS		T^2^	H^3^	T×H^4^
Total SCFA [mM]	111	124	114	4.85	0.14	0.55	0.84
SCFA							
Acetate [A]	64.1	69.2	68.8	2.88	0.31	0.70	0.75
Propionate [P]	21.4	24.5	19.8	1.91	0.23	0.44	0.95
Butyrate	18.7	22.8	18.9	2.66	0.47	0.84	0.99
Iso-butyrate	0.90^c^	1.10^b^	1.20^a^	0.14	0.04	0.21	0.93
Iso-valerate	4.80^b^	5.50^a^	3.40^c^	0.54	0.03	0.99	0.99
Valerate	1.25^-^	1.21^-^	1.41^-^	0.07	0.11	0.77	0.41
A:P ratio	3.72^-^	3.75^-^	3.69^-^	0.30	0.99	0.87	0.99

^1^SEM = standard error of the mean

^2^T: Treatments

^3^H: Hour collected

^4^TxH: interaction between treatments and hour collected

^abc^ means whit superscripts in each row differ by Tukey test (α = 0,05).

The animals fed MOS in this study presented the highest concentration of isobutyrate in the rumen, followed, in order, by those fed Yeast and the Controls (P<0.05). For isovalerate, the highest value was observed in animals fed Yeast, followed by Controls, then MOS (P<0.02). The concentration of valerate showed a trend (P = 0.11) to increase with the inclusion of MOS in the animals' diet. Iso-acids (isovalerate and isobutyrate) and valerate are formed during the fermentation of amino acids. Lascano and Heinrichs [32] concluded that supplementation with Yeast may stimulate the growth of amylolytic bacteria, which preferentially use peptides and amino acids due to their proteolytic activity, resulting in higher ruminal concentrations of iso-acids and valerate.

The diet in the current experiment was formulated with 90.6% of ground corn, aiming to challenge the rumen function. Supplementation with Yeast or MOS has led to a higher mean ruminal pH when compared to the Control treatment (P<0.01; Table 4).

**Table 4:** Ruminal pH and ammonia concentration in cattle fed grain-based diets supplemented with Yeast (live yeast *S*. *cerevisiae*) and MOS (mannan-oligosaccharides).

Item	Treatments			SEM^1^	P- value
	Control	Yeast	MOS		
Ruminal pH ruminal					
Mean ruminal pH^2^	5.90^b^	6.03^a^	6.13^a^	0.05	0.02
Maximum ruminal pH	6.52	6.71	6.39	0.06	0.76
Minimum ruminal pH	5.21	5.62	5.56	0.08	0.21
pH ≤5.8					
Duration h/d	10.66	5.66	5.00	2.44	0.38
AUC^3^, pH 5.8; pH x h/d	2.37	1.24	1.17	0.57	0.42
Ammonia, NH_3_ [mg/dL]					
Mean NH_3_ [mg/dL]	16.47^a^	14.47^b^	13.43^b^	1.82	0.02
Maximum NH_3_	41.60	39.41	37.35	3.45	0.72
Minimum NH_3_	2.05	2.49	2.49	0.77	0.84

^1^SEM = standard error of the mean

^2^mean value during the period of 0 to 21 h after the first feeding

^3^AUC = area under the curve

^ab^means whit superscripts in each row differ by Tukey test (α = 0,05).

Studies conducted by Gozho et al. [33] and Zebeli et al. [34] suggest that ruminal pH below 5.8 for more than 5.4 h/d is an indicator of subacute ruminal acidosis. Bach et al. [35] observed a significant increase in the feeding frequency of animals supplemented with Yeast, which may explain in part the stabilization of rumen pH and its consequent improvement found in this study. Additionally, the effects of *S*. *cerevisiae* and MOS on the stability of ruminal pH have also been attributed a reduction in the concentration of ruminal lactic acid because of the elevated growth of bacteria, such as *Megasphaera elsdenii* and *Selenomonas ruminantium*, the main types of lactate-utilizing bacteria [7, 36].

The effect of Yeast on ruminal microorganisms has been attributed to the ability of the yeast to capture oxygen, toxic to rumen bacteria, thereby improving bacterial growth within the rumen [37, 38]. On the other hand, the use of live yeasts *in vitro* studies has demonstrated that they can act by providing dicarboxylic acids (particularly malic acid), pro-vitamins and micronutrients, favoring the growth of anaerobic ruminal bacteria [39]. The mechanisms of action of MOS on pH regulation and growth of ruminal microorganisms have not yet been examined in detail, however this effect has been observed by several authors as Lei et al. [8], Diaz et al. [10], Li et al. [40] and Li et al. [41].

Animals fed the MOS diet had a lower ruminal NH_3_ concentration (P<0.01) than those fed the Control diet but did not differ from (P>0.05) the animals that received Yeast (Table 4). According to Tripathi and Karim [42], changes in the metabolism of N in the presence of yeast are due to the inhibitory activity of rumen proteolytic bacteria that limit their action on proteins and peptides [7]. However, Kumar et al. [43] asserted that this reduction may not be associated with decreased dietary protein degradation or deamination [44] but appears to be related to increased NH_3_ uptake into microbial protein, better profile of amino acids from duodenal digesta [7] and increases in the flow of bacterial nitrogen in the small intestine [42]. A reduction in NH_3_ concentration is also associated with reduced N excretion and energy expenditure for the synthesis and excretion of urea resulting from metabolism [45].

### Lipopolysaccharides and acute phase proteins

Diets containing a high proportion of concentrates can cause a drop in rumen pH, and consequently the death of low pH-sensitive bacteria [46]. For Gram-negative bacteria, this situation triggers the release of cell wall components, such as LPS [47, 48]. These compounds are toxic to animals and can induce local inflammation, which increases the permeability of the ruminal wall [13, 49] and adversely affects the gastrointestinal barrier, causing the translocation of LPS from the gastrointestinal tract into the bloodstream [13, 48].

In the current study, the mean concentrations of LPS in the plasma, rumen fluid, and duodenal digesta were 0.67, 52,457 and 49,035 EU/mL, respectively (Table 5). Similar results (LPS of 0.77 EU/mL in plasma, 42,418 EU/mL in rumen fluid, and 40,509 EU/mL in duodenal digesta) were observed by Lei et al. [8] in beef cattle fed a grain-based diet.

**Table 5:** Lipopolysaccharides (LPS) in rumen, duodenum, and plasma of cattle fed grain-based diets supplemented with Yeast (live yeast *S*. *cerevisiae*) and MOS (mannan-oligosaccharides).

LPS[EU^1^/mL]	Treatments			Mean	SEM^2^	P- value		
	Control	Yeast	MOS			T^3^	H^4^	T×H^5^
Rumen								
0 h	55,118	57,998	54,806	55,974	2,411	0.38	0.22	0.60
6 h	52,566	52,650	53,025	52,747				
12 h	50,635	51,250	44,063	48,649				
Mean	52,773	53,966	50,637					
Duodenum								
0 h	51,283	53,551	41,390	48,741	5,725	0.24	0.86	0.97
6 h	55,130	51,110	45,820	50,686				
12 h	52,635	46,913	43,490	47,679				
Mean	53,016	50,524	43,566					
Plasma								
0 h	0.77	0.63	0.62	0.67	0.017	0.04	0.28	0.53
6 h	0.73	0.71	0.64	0.69				
12 h	0.75	0.61	0.54	0.63				
Mean	0.75^a^	0.65^b^	0.60^b^					

^1^EU: endotoxins unit

^2^SEM = standard error of the mean

^3^T Treatments

^4^H: Hour collected

^5^TxH: interaction between treatments and hour collected

^ab^means whit superscripts in each row differ by Tukey test (α = 0,05).

By inducing subacute ruminal acidosis in dairy cows, Khafipour et al. [14] obtained increases of free LPS in the rumen from 28,184 to 107,152 EU/mL and in the plasma from 0.05 to 0.81 EU/mL, measured12 h after feeding. According to Gozho et al. [33], grain-rich diets increase the concentration of LPS in the rumen and its translocation into the plasma. The magnitude of the response depends on the level of concentrate in the diet and on the length of the feeding period.

The additives did not influence (P>0.05) the concentrations of LPS in the rumen or duodenal digesta (Table 5). However, they dramatically reduced LPS concentrations (P<0.01) in the plasma. This outcome may be attributed to the presence of the β-d-glucans involved in structuring the yeast cell wall, which are capable of recognizing and binding bacterial toxins, decreasing the absorption of these toxic substances [8, 50]. Lei et al. [8] found that dietary supplementation with MOS in cattle lowered plasma LPS concentration from 0.96 (control) to 0.66 EU/mL (with MOS). Diaz et al. [10] found that plasma LPS concentration reduced from 0.94 (control) to 0.46 and 0.44 EU/mL with Yeast and MOS, respectively, in sheep fed high grain diets. The MOS-LPS binding process occurs via electrostatic attraction between the positive charges on the yeast cell wall and the negative ligands present in the endotoxins [8].

Lynch and Martin [51] verified that Yeast and MOS have a regulatory effect on ruminal pH *in vitro*, reducing the death of Gram-negative bacteria in the rumen. Therefore, a dual role may be suggested for these additives, that is, as free LPS binders and as reducers of LPS release in the rumen. However, in the present study, although there was an increase in ruminal pH with the additives (Table 4), rumen LPS concentrations were not reduced (P>0.05; Table 5).

The blood levels of acute phase proteins (APP), SAA, and Hp were consistent with values when animals develop subacute ruminal acidosis due to high grain intake [10, 50]. The baseline levels of SAA and Hp in cattle are <25 and <50 μg/mL, respectively [51], and therefore the values found in this study are above normal (acute inflammatory response; Table 6).

**Table 6:** Serum amyloid A (SAA) and haptoglobin (Hp) in serum of cattle fed grain-based diets supplemented with Yeast (live yeast *S*. *cerevisiae*) and MOS (mannan-oligosaccharides).

Item	Treatments			Mean	SEM^1^	P- value		
	Control	Yeast	MOS			T^2^	H^3^	T×H^4^
SAA [μg/mL]								
0 h	32.77	33.44	29.61	31.94^a^	0.86	0.02	0.05	0.53
6 h	36.22	30.39	28.38	31.66^a^				
12 h	31.39	25.05	25.22	27.22^b^				
Mean	33.46^a^	29.63^b^	27.74^b^					
Hp [μg/mL]								
0 h	350.8	323.8	312.6	329.1	16.15	0.83	0.34	0.14
6 h	302.4	357.7	323.9	328.0				
12 h	325.0	258.0	321.3	301.4				
Mean	326.1	313.2	319.3					

^1^SEM = standard error of the mean

^2^Treatments

^3^H: Hour collected

^4^TxH: interaction between treatments and hour collected

^ab^means whit superscripts in each row differ by Tukey test (α = 0,05).

The increase in APP is related to the presence of LPS in the systemic circulation, which stimulates the release of proinflammatory cytokines, such as tumor necrosis factor-alpha (α-TNF), and interleukins 1 and 6 (IL-1 and IL- 6), by macrophages in the liver [48]. In addition, these macrophages activate hepatic receptors and initiate the synthesis of APP such as SAA and Hp, which are also used as markers of acute inflammatory response [46, 52].

Plasma concentrations of SAA were lower in the animals (P = 0.02) fed Yeast and MOS when compared to the animals that received the Control diet. There was no treatment × hour interaction (P>0.05). However, the concentrations of this protein decreased (P = 0.05) over the time of the sample collection period. This may have been due to the presence of β-glucans and mannan in the yeast cell wall, which may have weakened the proinflammatory immune response through the production of anti-inflammatory cytokines and the transient decrease in the expression of toll-like receptor 4, a component involved in the production of proinflammatory cytokines and acute phase proteins [53].

Similarly, the reduction in SAA concentration accompanying supplementation with *S*. *cerevisiae* probably occurred because of its stabilizing effect on ruminal pH, since this parameter is related to the release of LPS. As ruminal pH increased, plasma levels of LPS were reduced, which may have decreased blood concentrations of SAA as well. However, there was no effect (P>0.83) on the blood concentrations of Hp over treatments or collection times, and hence there was no interaction between these factors.

### Hematology

All erythrogram parameters were within the normal range [54] and were not significantly different (P>0.05) across treatments. In the leukogram, the total counts of leukocytes and lymphocytes were above the reference values for bovines (reference values: 4000–12,000 and 2500–7500 cells/μL respectively [44] (Table 7). Similarly, the number of segmented neutrophils was slightly above the reference value (600–4000 cells/μL) [54].

**Table 7:** Hematological results of cattle fed grain-based diets supplemented with Yeast (live yeast *S*. *cerevisiae*) and MOS (mannan-oligosaccharides).

Item	Treatments			SEM^1^	P- value
	Control	Yeast	MOS		
Erythrogram					
Erythrocytes [10^6^/μL]	6.9	7.0	6.7	0.28	0.74
Hemoglobin [g/dL]	9.8	10.4	9.8	0.25	0.61
Hematocrit [%]	26.3	27.6	26.0	0.68	0.65
M.C.V^2^ [fl]	38.2	39.3	38.7	1.28	0.61
M.C.H.C^3^ (%]	37.2	37.8	37.6	0.12	0.16
Platelets [10^3^/μL]	595.6	421.0	469.6	39.3	0.17
Leukogram					
Leukocytes [cells/μL]	15,260	16,060	15,293	612.1	0.80
Segmented Neutrophils [cells/μL]	4,105	4,406	4,246	652.3	0.33
Lymphocytes [cells/μL]	9,341	8,960	10,377	516.9	0.48
Monocytes [cells/μL]	593	686	933	136.4	0.34
Eosinophils [cells/μL]	938	1,608	666	307.2	0.51

^1^SEM: standard error of the mean.

^2^M.C.V: Mean Corpuscular Volume

^3^M.C.H.C: Mean Corpuscular Hemoglobin Concentration. Tukey test (α = 0.05).

The leukocytosis and lymphocytosis (relative to reference values) reported in this study may have occurred because of the increased grain in the diet which increases the release of LPS into the rumen and its translocation into the bloodstream, causing inflammatory stimulation [33] and promoting the increase in the levels of APP (SAA and Hp) above the reference values for the bovines. In fact, neutrophils, which are the defense cells of the body and responsible for the phagocytosis of pathogens, are involved in the synthesis and release of cytokines, thus protecting the body from bacterial challenge. For this reason, increased concentrations of segmented neutrophils may be observed during inflammatory processes as well [55].

## Conclusion

The inclusion of 1.5 g/kg DM of Yeast and 1.5 g/kg DM of MOS in high-grain diets for finishing cattle reduces NH_3_ concentration and increases the pH and total concentration of SCFAs in the ruminal fluid. The additives also decreased plasma LPS and SAA concentrations, contributing to the reduction of the inflammatory process in the animals caused by consumption of the grain-based diets.

Although additives such as Yeast and MOS have different characteristics and modes of action, they yielded similar results in this study, indicating that both can be used to improve the rumen environment of beef cattle susceptible to subacute ruminal acidosis.

## Supporting information

S1 Fig Ruminal pH of Holstein steers fed high concentrate diets supplemented with Yeast (live yeast *Saccharomyces cerevisiae)* and mannan-oligosaccharides (MOS). (XLSX)

S2 Fig Ammonia (NH_3_) of Holstein steers fed high concentrate diets supplemented with Yeast (live yeast *Saccharomyces cerevisiae)* and mannan-oligosaccharides (MOS). (XLSX)
